# Supplementary material for: Role and mechanism of NCAPD3 in promoting malignant behaviors in gastric cancer
Source: Front Pharmacol. 2024 Apr 22;15:1341039. doi: 10.3389/fphar.2024.1341039 (PMC11070777; doi:10.3389/fphar.2024.1341039)
Supplement: Supplementary file 11 [file DataSheet2.ZIP › GSEA/Oncogenic signature/my_analysis.Gsea.1599461962066/RAF_UP.V1_DN.html]

Details for gene set RAF\_UP.V1\_DN[GSEA]

|  || Dataset | filtered\_dataset.sample\_info.cls#WT\_versus\_NCAPD3\_MUT |
| Phenotype | sample\_info.cls#WT\_versus\_NCAPD3\_MUT |
| Upregulated in class | NCAPD3\_MUT |
| GeneSet | RAF\_UP.V1\_DN |
| Enrichment Score (ES) | -0.42612985 |
| Normalized Enrichment Score (NES) | -1.9576132 |
| Nominal p-value | 0.006833713 |
| FDR q-value | 0.029136732 |
| FWER p-Value | 0.164 |
Table: GSEA Results Summary

  

Fig 1: Enrichment plot: RAF\_UP.V1\_DN      
 Profile of the Running ES Score & Positions of GeneSet Members on the Rank Ordered List

  

| SYMBOL | TITLE | RANK IN GENE LIST | RANK METRIC SCORE | RUNNING ES | CORE ENRICHMENT || 1 | 323 | APBB2 | 35 | 0.977 | 0.0551 | No |
| 2 | 3667 | IRS1 | 83 | 0.821 | 0.0887 | No |
| 3 | 8573 | CASK | 368 | 0.554 | -0.0700 | No |
| 4 | 2188 | FANCF | 545 | 0.449 | -0.1598 | No |
| 5 | 11083 | DIDO1 | 631 | 0.406 | -0.1876 | No |
| 6 | 4071 | TM4SF1 | 851 | -0.251 | -0.3245 | No |
| 7 | 2037 | EPB41L2 | 943 | -0.365 | -0.3599 | No |
| 8 | 9926 | LPGAT1 | 1036 | -0.434 | -0.3904 | Yes |
| 9 | 8030 | CCDC6 | 1081 | -0.462 | -0.3841 | Yes |
| 10 | 857 | CAV1 | 1098 | -0.475 | -0.3566 | Yes |
| 11 | 8727 | CTNNAL1 | 1106 | -0.482 | -0.3221 | Yes |
| 12 | 26053 | AUTS2 | 1113 | -0.485 | -0.2865 | Yes |
| 13 | 54541 | DDIT4 | 1133 | -0.502 | -0.2590 | Yes |
| 14 | 6947 | TCN1 | 1135 | -0.503 | -0.2184 | Yes |
| 15 | 27250 | PDCD4 | 1170 | -0.537 | -0.1987 | Yes |
| 16 | 4651 | MYO10 | 1213 | -0.588 | -0.1806 | Yes |
| 17 | 26130 | GAPVD1 | 1247 | -0.625 | -0.1530 | Yes |
| 18 | 202 | AIM1 | 1290 | -0.681 | -0.1273 | Yes |
| 19 | 5046 | PCSK6 | 1297 | -0.699 | -0.0742 | Yes |
| 20 | 7031 | TFF1 | 1309 | -0.713 | -0.0235 | Yes |
| 21 | 1475 | CSTA | 1400 | -1.162 | 0.0072 | Yes |
Table: GSEA details [plain text format]

  

Fig 2: RAF\_UP.V1\_DN      
 Blue-Pink O' Gram in the Space of the Analyzed GeneSet

  

Fig 3: RAF\_UP.V1\_DN: Random ES distribution      
 Gene set null distribution of ES for **RAF\_UP.V1\_DN**
